# Proteins and amino acids are fundamental to optimal nutrition support in critically ill patients

**DOI:** 10.1186/s13054-014-0591-0

**Published:** 2014-11-17

**Authors:** Peter JM Weijs, Luc Cynober, Mark DeLegge, Georg Kreymann, Jan Wernerman, Robert R Wolfe

**Affiliations:** Department of Nutrition and Dietetics, Internal Medicine, VU University Medical Center Amsterdam, De Boelelaan 1117, 1081 HV Amsterdam, the Netherlands; Department of Intensive Care Medicine, VU University Medical Center Amsterdam, De Boelelaan 1117, 1081 HV Amsterdam, the Netherlands; Department of Nutrition and Dietetics, Amsterdam University of Applied Sciences, Wibautstraat 2-4 1091 GM, Amsterdam, the Netherlands; EMGO+ Institute of Health and Care Research, VU University Medical Center Amsterdam, De Boelelaan 1117, 1081 HV Amsterdam, the Netherlands; Clinical Chemistry Department, Cochin and Hôtel-Dieu Hospitals, APHP, 1 place du Parvis Notre-Dame 75004, Paris, France; Nutrition Lab, EA 4466, Department of Experimental, Metabolic and Clinical Biology, Faculty of Pharmacy, Paris Descartes University, 12 rue de l’Ecole de Médicine 75270, Paris, France; Baxter Healthcare, Deerfield, IL 60015-4625 USA; Baxter Healthcare SA Europe, CH-8010 Zürich, Switzerland; Department of Anesthesiology and Intensive Care Medicine, Karolinska University Hospital, Huddinge, Karolinska Institutet, 141 86 Stockholm, Sweden; Center for Translational Research in Aging & Longevity, Department of Health & Kinesiology, Texas A&M University, 4243 Ireland St #336, College Station, TX 77843 USA

## Abstract

Proteins and amino acids are widely considered to be subcomponents in nutritional support. However, proteins and amino acids are fundamental to recovery and survival, not only for their ability to preserve active tissue (protein) mass but also for a variety of other functions. Understanding the optimal amount of protein intake during nutritional support is therefore fundamental to appropriate clinical care. Although the body adapts in some ways to starvation, metabolic stress in patients causes increased protein turnover and loss of lean body mass. In this review, we present the growing scientific evidence showing the importance of protein and amino acid provision in nutritional support and their impact on preservation of muscle mass and patient outcomes. Studies identifying optimal dosing for proteins and amino acids are not currently available. We discuss the challenges physicians face in administering the optimal amount of protein and amino acids. We present protein-related nutrition concepts, including adaptation to starvation and stress, anabolic resistance, and potential adverse effects of amino acid provision. We describe the methods for assessment of protein status, and outcomes related to protein nutritional support for critically ill patients. The identification of a protein target for individual critically ill patients is crucial for outcomes, particularly for specific subpopulations, such as obese and older patients. Additional research is urgently needed to address these issues.

## Introduction

Proteins and amino acids (AAs) play a major role in the maintenance of body homeostasis and their metabolism in critically ill patients has been intensively researched over the past 40 years. In contrast, few studies of optimal protein or AA intake have been conducted in patients requiring nutritional support [1]. This review highlights the importance of protein in nutritional support for critically ill patients and provides the rationale for conducting additional, much-needed studies to improve protein and AA nutritional support.

In this review, protein intake refers to enteral nutrition, AA intake refers to parenteral nutrition (PN), and nitrogen (N) generally refers to metabolism and overall N balance. The administration of AA at pharmacological levels (for example, glutamine, arginine) is not discussed in detail because a number of reviews and meta-analyses have been published recently on this topic [2-4].

## A historical perspective

Protein nutrition, provided by intravenous AA infusion, has been used for more than 70 years [5] and it was believed that insufficient intravenous energy provision prevented the efficient use of these AAs [6]. Infusion of hypertonic glucose through a central venous catheter was first described in 1955 [7] and indwelling catheters, hypertonic glucose, protein hydrolysates, and other nutrients were used successfully on a limited clinical basis over the next 10 years. Dudrick and colleagues demonstrated that all nutrients necessary for growth could be provided by intravenous feeding [8]. Over the following decade, research focused on accurately defining nutritional requirements, particularly for micronutrients, and on the technical aspects of adequate intravenous delivery of energy or AAs. As it became technically possible to deliver more nonprotein energy intravenously, the problem of overfeeding arose. One adverse effect of overfeeding is the deposition of fat in the liver, particularly in patients receiving total PN for reversal of the catabolic response to critical illness [9,10].

The progressive and rapid loss of body mass and muscle well known to occur in patients with critical illness was termed hypermetabolism. The view at the time was that critically ill patients have very high energy expenditure and therefore require high-energy nutrition. Septic autocannibalism was a term used to describe the loss of muscle mass that does not benefit from increasing AA provision above minimum requirements [11]. Therefore, through the 1970s, researchers focused on ensuring that energy intake exceeded expenditure, rather than on targets for adequate protein intake [9]. Later, when enteral nutrition became a viable option in critically ill patients, nutritional interventions continued to focus on meeting energy requirements. When recommendations for protein or AA intake were given, they were generally expressed as a function of energy intake. For example, in the 1990s the American College of Chest Physicians recommended that, for patients in ICUs, ‘15-20% of the total calories administered per day can be given as protein or amino acids’ [10]. However, the guidelines provided neither the rationale nor the scientific basis for this recommendation.

As we move further into the 21st century, our understanding of the importance of protein nutrition in critically ill patients is increasing. However, further research is needed to better define the optimal intake of protein, in the same way that total energy requirements are defined at present.

## Important protein-related concepts: a metabolic perspective

### Adaptation to starvation

In a healthy person, an inadequate supply of protein and energy (that is, semi-starvation or full starvation) results in protein energy malnutrition. Semi-starvation induces metabolic adaptations in protein metabolism that limit the rate at which lean mass is lost [12,13]. When energy supplies fall short in a healthy person, physical activity is reduced to maintain an energy balance. However, a decrease in activity to some extent counteracts the adaptations that limit the loss of lean mass. During starvation, heart and lung muscle may initially be preferentially spared and a disproportionate amount of peripheral skeletal muscle is lost. Ultimately, however, the essential muscle mass of vital organs will also be reduced and, in combination with underused peripheral skeletal muscle, results in muscle weakness and functional disability [12,13]. These adaptive responses may be considered successful because they allow humans to survive limited periods of starvation. However, if protein and energy provisions continue to remain inadequate, essential bodily functions, such as the immune system, will be adversely affected. Impaired immune function may lead to an increased risk of infections, which in starvation is often seen as a direct cause of death. Starvation has serious implications for a healthy individual, which are even more pronounced in critically ill patients, especially those with a low body mass index [14].

### Adaptation of protein and amino acid metabolism under conditions of stress

Humans have developed survival systems during metabolic stress, such as AA use for gluconeogenesis and acute phase protein reactions, increased protein turnover, and adapted regulation by catabolic hormones. In stress situations, the priority of the metabolic response is to provide energy to both the brain and injured tissues to promote healing [15,16]. Humans have very limited glucose stores and cannot synthesize glucose from fat. Therefore, in the absence of glucose intake, glucose is synthesized from gluconeogenic AA, lactate, and pyruvate. In the starving healthy individual, glucose infusion inhibits gluconeogenesis. In stress situations, however, gluconeogenesis is not readily reversed by glucose infusion, implying that N may be lost through ureagenesis. The pool of free essential AAs is very small, with most generated from net proteolysis, occurring particularly within muscles [17]. In critically ill patients, in parallel with the severity of the injury, increases in proinflammatory cytokines, glucocorticoids, and oxidative stress reinforce the effect of catabolic hormones, and contribute to insulin resistance and muscle wasting [18,19]. Insulin resistance is common in critically ill patients [20], and contributes to net muscle protein catabolism and liver gluconeogenesis (see Anabolic resistance).

In critically ill patients, a fraction of the essential AAs arising from protein breakdown is oxidized in muscle at an increased rate, in particular branched-chain AAs, while another fraction is released into the blood at an accelerated rate and rapidly cleared by organs such as the liver. In general, there is a unidirectional flux of all AAs from muscle to liver, controlled by hormones. Cortisol promotes net muscle proteolysis and AA release, while glucagon promotes AA uptake by the liver and AA use in gluconeogenesis [21]. In the liver there is a large increase in AA uptake for gluconeogenesis and for protein synthesis, including the synthesis of acute phase proteins [22,23]. Some AAs are also taken up selectively by other tissues for specific purposes. For example, glutamine is taken up by the kidneys (to sustain ammoniagenesis and counteract acidosis), by fibroblasts and enterocytes (for healing), and by immune cells (for replication and action) [24].

As long as these processes remain adaptive, plasma AA levels remain stable and in balance. However, when the stress response becomes too intense and persists for too long, the balanced AA profile in plasma is disrupted, resulting in abnormal AA concentrations (both higher and lower levels than normal) [25-27]. In particular, plasma glutamine concentrations have been associated with unfavorable outcomes in critically ill patients [28,29].

Under stress conditions, the various alterations in protein metabolism are reflected by an increase in whole-body protein turnover [30,31] and an increase in AA oxidation and N loss. Since all proteins within the body have specific functions (see Table 1), they cannot be considered a form of AA storage, highlighting the importance of exogenous sources of protein or AA by nutritional support.

**Table 1:** **Functions of proteins**

•	Proteins are the major components of muscles, required for muscle dynamics and function
•	Enzymes are proteins. Therefore, proteins are essential for intermediary metabolism and energy production. Similarly, all cell carriers are proteins
•	Some proteins are involved in specific immunity (that is, immunoglobulins) and in nonspecific immunity (for example, opsonins)
•	Proteins contribute to the architecture and structure of organs and tissues. A typical example is collagen, which has a major architectural role, for example, in bone and skin
•	Proteins secreted into the blood by the liver are carriers of lipid-soluble molecules: hormones (for example, transthyretin for thyroxin), vitamins (for example, retinol binding protein for vitamin A), nutrients (for example, albumin for free fatty acids and tryptophan), and a number of drugs
•	Proteins in the blood, especially albumin, are involved in the control of oncotic (colloid osmotic) pressure
•	Proteins contribute physiologically to energy expenditure (12 to 15% of total daily expenditure) in the postabsorptive state, through release of amino acids following proteolysis. This may occur directly (for example, branched-chain amino acids in the muscles) or indirectly (through glucose (gluconeogenesis) or ketone body (ketogenesis))

### Muscle proteins

Muscle proteins are in a constant state of turnover and the balance between the rates of protein breakdown and synthesis determines whether there is a net gain (anabolism) or a net loss (catabolism, wasting). A number of circumstances (for example, burn injury, trauma complicated with sepsis, head trauma) result in loss of muscle mass driven by an accelerated rate of protein breakdown [32]. The increased release of AAs from protein breakdown provides a stimulus for accelerated muscle protein synthesis [33]. Protein synthesis cannot match the increased rate of muscle protein breakdown, although protein synthesis rates do vary significantly from patient to patient [22,34].

In a stress situation, the catabolic loss of muscle can be avoided only if the uptake of AAs from the blood is increased either by intravenous infusion or the digestion of enterally administered proteins, peptides, or AAs. These sources of AA may then stimulate protein synthesis to offset the accelerated rate of protein breakdown and AA oxidation [22,35,36].

### Anabolic resistance

Anabolic resistance refers to the state in which a patient is resistant to the normal stimulatory effect of AAs on muscle protein synthesis. This is common in older patients and may contribute to the estimated 1.4 to 2.5% muscle loss per year in that group [37]. Anabolic resistance is also frequently observed in critically ill patients [38], in patients on bed rest, or in people exposed to weightless environments (such as in space) [39]. Three main factors explain anabolic resistance: splanchnic sequestration of AAs following feeding [40,41], which decreases the AAs available to muscles; insulin resistance, which limits AA uptake into muscles [42] and hinders the maintenance of muscle protein [39]; and blunted responses to AAs with anabolic properties, such as the essential AA leucine [19,43]. The older patients require a higher intake of essential AAs compared with the young to generate the same acute response [42]. However, there is a lack of data on AA requirements in older patients in the ICU; these requirements warrant urgent research, since the number of older patients in the ICU is likely to increase in parallel with that of the general population. Muscle protein anabolism can be optimized in otherwise healthy individuals by a combination of adequate protein or AA intake, combined with exercise and insulin [44].

Anabolic resistance is also driven by insensitivity to the normal anabolic action of leucine. In stressed patients, the level of muscle-free leucine is higher than in patients without stress [25,45,46]. Experimental models show that leucine stimulates protein synthesis via signaling through the mammalian target of rapamycin system [47]. Leucine and insulin share common pathways for activating protein synthesis, suggesting that signal transduction is impaired somewhere within the mammalian target of rapamycin pathway. However, there may be differences in the acute and prolonged effects of leucine supplementation [48]. Research into the role of leucine in different clinical settings has potential implications for the treatment of muscle loss in metabolically stressed patients.

### Interaction of protein with nonprotein energy

The efficiency of protein intake in stimulating protein synthesis is dependent to some extent on the level and nature of the nonprotein energy intake. Many nutritional guidelines for macronutrient intake during nutritional support are derived from requirements for healthy individuals. It has been known for almost 100 years that the amount of protein intake required to maintain N balance in healthy individuals is a function of the amount of concomitant energy intake [49]. Meeting energy requirements is a major goal of appropriate nutrition support. However, delivering excessive energy to severely stressed patients usually fails to enhance the retention of body protein [50] and often causes adverse effects, such as severe fatty infiltration of the liver [51]. In a catabolic state, loss of lean body mass (LBM) often cannot be entirely prevented by nutrition support.

The realistic aim of nutrition support is to blunt the loss of LBM, and the goal should be to supply the amount and nature of protein or AA intake that can maximally stimulate protein synthesis [52]. Even in the insulin-resistant state, AAs can stimulate, to some extent, muscle protein synthesis [53]. The provision of adequate, but not excessive, nonprotein energy may provide some benefit to stressed patients. The potential for detrimental effects is considerable, however, and care should be taken to avoid supplying nonprotein energy in excess of energy expenditure.

### Amino acid transport

The first step in AA processing is cellular uptake, and many different AA transporters occur in different cell types [54]. There are also transporters that cycle certain AAs between the cytosol and mitochondria. For example, the ureagenesis pathway transports ornithine from the cytosol to mitcohondria and citrulline from mitochondria to the cytosol [55]. One transporter may take up several AAs, and a single AA may have more than one transporter. Consequently, some AAs compete with each other for cellular uptake and the provision of an imbalanced AA solution may lead to an inappropriate cellular response. The pharmacokinetic properties of any new AA formula should therefore be tested, as well as the quantity and quality of the AA formulation.

### Potential adverse effects of amino acid provision

Most AAs are precursors of neuromediators or of false neuromediators (that is, AAs or metabolites able to bind to neuroreceptors, eliciting or blocking the messages of the true mediators). Administration of large nonphysiologic amounts of AA may lead to seizures and brain damage [56]. Defining an upper limit of safe intake for each AA is therefore of utmost importance [57]. This has been achieved for leucine, which has an upper limit of safe intake of 0.53 g/kg body weight/day in healthy subjects [58]. Of note, this level of leucine intake is well above the normal requirement. There is currently no recommendation concerning the safety of leucine administration in patients, due to the lack of dose-ranging studies. Tolerability varies from one AA to another [59]. Some AAs appear extremely safe even when given in the 15 to 30 g/day range (for example, glutamine, citrulline) [24], at least in the short term [60], whereas tolerance to other AAs (for example, methionine) seems limited [61]. At present, the safety of arginine in ICU patients is a topic of some debate [62,63].

## Methods for assessment of protein status

### Nitrogen balance

The N balance is commonly used as the basis for estimating protein requirements in healthy individuals [64,65] and in patients [66-68]. The N balance becomes negative when intake falls short of the minimum protein requirement, leading to loss of LBM. In an otherwise healthy person, however, a chronic low-protein diet may lead to adaptation to a stable overall N balance [69].

Estimation of both N intake and N loss presents challenges that must be considered carefully [70]. While N intake can be accurately measured in patients supported with total enteral nutrition or PN, the assessment of oral dietary protein intake is extremely difficult in clinical practice. Measuring N loss is also problematic, because it is not easy to collect complete 24-hour urine samples, especially in patients with large fecal or urinary output (for example, those with burns or a bone marrow transplant) [71]. In the critically ill patient, total body N loss from urinary urea content underestimates N loss as these patients have an increase in ammonia loss not accounted for in N calculations from urinary urea [72,73]. Therefore, in ICU patients, total urinary N loss should be measured directly rather than estimated from urinary urea. However, even direct measurements of urinary loss do not reflect the total loss, as they do not include N loss from diarrhea, fistulas, or draining wounds [71].

A positive N balance is not synonymous with improved protein balance, as the N balance after administration of a load of a single AA may simply reflect cellular accumulation of the free AA [74]. In addition, for critically ill patients, intravenous administration of protein-containing blood products confounds N-balance assessments. Nevertheless, measurement of urinary N loss and calculation of N balance remains the most commonly used biomarker to assess protein accretion or loss in ICU patients. Additional information on plasma and urinary levels of conventional markers of protein status is provided in Table 2.

**Table 2:** **Plasma and urinary levels of conventional markers of protein status**

**Subject of measurement**	**Rationale**	**Usage**
Plasma protein levels: albumin, transthyretin (formerly called prealbumin), and retinol binding protein	These proteins are selectively synthesized by the liver. Therefore, it is generally believed that their rate of synthesis parallels the supply of amino acids. In the case of inflammation, plasma levels of these proteins do not indicate nutritional status	Transthyretin measurements can be used to assess the efficacy of nutrition support [75], while albumin measurements can be used to assess the risk of complications associated with malnutrition. When used for this purpose, albumin may be used alone [76] or, ideally, as an index to be considered in combination with variations in body weight over time (nutritional risk index [77] in adults, geriatric nutritional risk index [78] in geriatric patients)
Urinary 3-methylhistidine (3MH)	3MH is derived from histidine with a post-transcriptional methylation at position 3. This amino acid is present mainly in myofibrillar proteins and, to a smaller extent, in intestinal smooth muscles. Following proteolysis, released 3MH is not reincorporated into proteins since there is no codon for this amino acid. Instead, 3MH is further eliminated into urine	There is a correlation between the 24-hour excretion of 3MH and myofibrillar proteolysis. Since the former will be dependent upon muscle mass, 3MH excretion must be expressed as a ratio to urinary creatinine. It has been clearly demonstrated that muscle myofibrillar proteins account for the entire increase in 3MH excretion during hypercatabolic states [79]. In chronic malnutrition, urinary 3MH is low due to restriction adaptation, and improvement in the nutritional state leads to an increase of this parameter because elevated protein synthesis leads to an increase in proteolysis
Plasma phenylalanine	Phenylalanine is mainly catabolized in the liver, and not in the muscle. The arteriovenous difference in phenylalanine concentration is a marker of muscle proteolysis. Unfortunately, arterial puncture is an invasive procedure, and is associated with technical problems that complicate the use of this marker. In addition, interpretation of the data requires that blood flow is measured simultaneously. Alternatively, plasma phenylalanine can be measured as a marker of protein turnover. Some authors have suggested measuring the phenylalanine:tyrosine ratio for this purpose	It has been shown [80] that plasma phenylalanine correlates well with nitrogen balance in burn patients. At present, there are insufficient data available to recommend the use of plasma phenylalanine or of the phenylalanine:tyrosine ratio as a reliable marker of protein turnover
Plasma citrulline	The amino acid citrulline is not included in proteins and it is almost absent in food. In the general circulation, most citrulline is formed in enterocytes and is mostly catabolized in the kidneys [81]. Of note, citrulline in the liver is strictly compartmentalized within periportal hepatocytes [61] and the liver neither takes citrulline up nor releases it, except in patients with liver cancer [82]	Following the pioneering work by Crenn and colleagues [83] in patients with short bowel syndrome, plasma citrulline has been validated as a marker of gut functional mass in a number of clinical situations (see [84] for a recent review)

### Assessment of qualitative and quantitative amino acid requirements

AA requirements may be assessed by simple pharmacokinetic studies. The balance between the requirement for and intake of an AA can be assessed by measuring the plasma concentration of that specific AA in the basal state and at various times during AA administration. Several studies, summarized in 2003 [85], indicate that during continuous parenteral administration of AAs there is a sharp increase in AA levels followed by a plateau that lasts several hours. The level of the plateau appears to be related to the rate of infusion of each AA [85]. It is therefore possible to construct a one-compartment model with first-order elimination kinetics [86] to study the relationship between the increase in plasma AA concentration and the rate of infusion for any AA. This model was tested in surgical ICU patients. In ICU patients receiving a more individualized AA solution, based on the dynamic test described above, abnormal changes in AA levels were almost eliminated, while they persisted in the control group. The 5-day N balance was significantly improved with this individualized kinetic approach in the surgical ICU study [87]. These studies suggest that, in the future, AA solutions for PN should be formulated for specific disease states. However, the concepts demonstrated by this kinetic approach will need to be validated by additional prospective clinical studies.

### Whole-body and muscle protein synthesis

Estimation of N balance is a black-box approach that provides no information about underlying mechanisms, such as variations of protein synthesis and breakdown at the whole-body or tissue level. An alternative approach is to quantify protein needs and determine appropriate protein provision by measuring protein synthesis in the whole body and/or muscle.

AA tracers are commonly used in clinical research. They are created by incorporating stable isotopes of carbon, hydrogen, or N into an AA to produce a uniquely identifiable molecule. Labeled AAs can be administered in small amounts by infusion or ingestion to trace the metabolism of the more abundant form of the AA in the body. Mass spectrometry analysis of the AA tracer in blood or protein enables the calculation of whole-body protein synthesis and whole-body protein breakdown. All proteins in the body, including muscle, will incorporate the labeled AA at rates that reflect the fractional synthetic rate of the protein [88].

The muscle fractional synthetic rate reflects only the synthetic rate of muscle protein. The net gain or loss of protein is determined by the balance between its rate of synthesis and its rate of breakdown. However, the muscle fractional protein breakdown rate is not commonly assessed because it requires a more complicated experimental protocol, additional blood sampling, and complex calculations, especially during feeding [89]. One approach to calculating muscle protein synthesis and breakdown is to measure the balance of specific AAs across the leg or arm [90]. This requires arterial and venous catheterization and measurement of tissue blood flow. When combined with muscle tissue biopsy data, the rates of muscle protein synthesis, breakdown, and net balance can be calculated. In addition, the rate of AA uptake and release into blood can be measured [90]. Differences in limb blood flow may alter the measured arteriovenous balance, however, limiting the applicability of this technique.

In a research setting, whole-body protein synthesis and breakdown can also be measured in response to protein feeding or PN. One commonly used approach is the addition of phenylalanine/leucine to a meal, together with an infusion of labeled tyrosine and phenylalanine, followed by blood sampling [88]. An advantage of this approach is that muscle biopsies are not required, and frequent blood sampling enables calculations of both synthesis and breakdown during protein or AA absorption. While tracer methods have been used to study the acute response of whole-body or protein synthesis in metabolically stressed patients, a systematic investigation of protein synthesis and breakdown has not yet been performed. An appropriately designed isotopic study providing direct evidence for the protein needs of critically ill patients would be of great value.

### Muscle mass and lean body mass

Measurement of LBM (that is, fat-free mass, including extracellular fluid) over time should provide important insights into an individual patient’s requirement for protein intake. However, attempts to measure LBM easily and accurately at the bedside in the ICU have to date been unsuccessful. It is important to determine which body-compartment measurement best reflects the daily amount of protein needed to maintain equilibrium. Total body mass is not useful because the varying amounts of fat mass are not relevant to protein metabolism. Also, standard body weight or ideal body weight measurements are not appropriate as they lead to imprecise estimates in obese patients [91]. The most appropriate compartments for determining protein requirements are LBM and body cell mass (that is, the sum of all living cells excluding extracellular fluid and bone mass). Both of these compartments can be measured in clinical practice using bioelectrical impedance analysis [92,93]. However, the overall precision of bioelectrical impedance analysis may be reduced by variations in hydration status. Dual-energy X-ray absorptiometry [94] can be used to assess LBM, although dual-energy X-ray absorptiometry scanners are not available in every hospital and are not routinely used for critically ill patients.

Computed tomography (CT) may also be used to assess LBM [95]. Specialized CT software has been used at the third lumbar spine to determine the area of skeletal muscle, as well as subcutaneous, intramuscular, and visceral adipose tissue. A lower LBM, as determined by CT imaging at the third lumbar spine, is an adverse prognostic factor in patients with pancreatic cancer and sarcopenic obesity [96]. Recently, Moisey and colleagues have demonstrated the prognostic value of LBM for predicting mortality in critically ill, older, sarcopenic, injured patients [97]. Weijs and colleagues confirmed these findings in a population with a much broader age range and mixed diagnoses [98].

Since CT scanning technology exists in most hospitals and many hospital patients undergo an abdominal CT scan, analysis of skeletal muscle in a single CT slice could be carried out routinely. However, routine use of CT scanning for patients in the ICU remains a difficult logistical problem. Bedside ultrasound imaging could also be used to measure muscle thickness, avoiding the potential issues associated with radiation doses from CT scans. However, additional research is needed to develop a reliable and sensitive technique for assessing LBM [99] and these methods require validation in this setting [68]. The report of muscle wasting in ICU patients by Puthucheary and colleagues highlights the importance of monitoring [34]. Recent studies have provided preliminary data showing a statistical relationship between muscle wasting and protein intake [100,101]. However, muscle mass varies too slowly to be used as a day-to-day tool for fine-tuning protein intake.

## Protein nutrition support and outcome in critically ill patients

### Muscle wasting and functional impairment

As outlined above, body protein from functional tissues is catabolized during critical illness and may culminate in a clinically significant loss of muscle mass [34]. The extent of muscle mass loss may impact the ultimate prognosis of an individual. Significantly reduced muscle mass often leads to impaired functioning, which hinders activities such as weaning from the ventilator and muscle function recovery. These adverse outcomes can result in increased nosocomial infection, such as pneumonia, bacteremia, and wound infection, leading to a renewed state of increased catabolism. This vicious cycle is associated with poor patient outcomes. Functional disability may be long term and may never fully return to normal levels [102,103]. The increased efflux of AAs from catabolic muscle also places a metabolic burden on the liver. Therefore, in highly catabolic states of muscle protein breakdown, it is clinically important that the liver clearance of AAs for protein synthesis and other metabolic functions remains intact [104,105].

Pharmacological therapy has the potential to work in concert with dietary protein to ameliorate the rate of muscle loss in critically ill patients. Among others, insulin [106], propranolol [107], and testosterone [108] have all been used successfully in seriously burned patients in conjunction with adequate nutritional support to lessen the extent of muscle protein loss. Pharmacological doses of growth hormone actually stimulate muscle protein synthesis in the critically ill [109]. However, critically ill patients treated with human growth hormone had a higher mortality than untreated patients [110]. In other words, blocking muscle protein breakdown is not recommended if adequate nutritional support is not provided. When feasible, the combination of adequate protein intake with early exercise should be further investigated as a means by which to improve both short-term and long-term outcomes.

### Level of protein nutrition support in critically ill patients

Current recommendations advise a protein intake level in critically ill patients of more than 1.2 g/kg/day [111-113]. Guidelines are based on results from studies employing N balance and body composition techniques in which protein intake exceeding 1.5 g/kg/day did not provide any advantage [15,111-114]. However, based on N balance data, authors of a systematic and critical review suggested that a level of 2.0 to 2.5 g/kg/day may be safe in many critically ill patients [68]. An observational study of patients with head trauma demonstrated that a higher N intake provided a better short-term N balance, which at best supports the conclusion of safety [115]. Further, a recent study evaluating whole-body protein turnover in adolescent patients given PN reported a protein balance advantage with a protein intake as high as 3.0 g/kg/day [116]. Whether this level of intake is appropriate for adults, however, is unknown. To date there have been no outcome studies providing this level of protein intake to critically ill patients. Even though recommended protein intakes for critically ill patients vary widely, there is a consistent recommendation that protein intake should exceed the levels of 0.8 g/kg/day required by normal, healthy individuals. At present, however, most critically ill adults receive less than one-half of the recommended protein intake (about 0.6 g/kg/day) [14].

### Current evidence for protein nutrition support and outcome

There appears to be a lower than recommended protein provision within the first week of patient admission [14,68]. The clinical outcomes of several studies reporting protein intake in critically ill patients are summarized in Table 3. Three large prospective trials [117-119] investigating the use of evidence-based feeding guidelines in critically ill patients showed no significant difference in mortality, while nonrandomized studies indicate a potential relationship between protein levels in nutrition support and mortality [14,120-122]. These studies suggest that protein supply is of major importance to outcome in critically ill patients.

**Table 3:** **Studies reporting protein intake in critically ill patients**

**Citation**	**Patient population**	**Study design**	**Clinical outcome: recovery, survival and length of stay**
Larsson and colleagues [114]	Severely injured patients (burn or fracture of more than two long bones). Randomized during the first week of trauma (*n* = 39) to five different amounts of N from 0 to 0.3 g/kg/day	Prospective randomized study	Daily and cumulative N balance increased in the groups with a N intake of up to 0.2 g/kg/day versus the no N group (*P* <0.001)
Ishibashi and colleagues [15]	Immediate post-trauma patients (*n* = 18) or severely septic patients (*n* = 5) were divided into three groups (A, B, and C) receiving 1.1, 1.5, and 1.9 g/kg FFMc/day protein respectively	Retrospective study	Average loss of total body protein was 1.2 kg. Loss of body protein was greater in group A compared with groups B (*P* = 0.013) and C (*P* = 0.023). Protein loss in group B (1.5 g/kg FFMc/day), was half that of group A (1.1 g/kg FFMc/day). Protein loss in groups B and C was not different. An intake of 1.5 g/kg FFMc/day was equivalent to 1.0 g/day/kg body weight measured at the start of the study. Authors recommend the clinician obtains information on pre-illness bodyweight and prescribes 1.2 g/day/kg
Barr and colleagues [118]	200 ICU patients (npo >48 hours after their admission): 100 before implementation of a nutritional management protocol, 100 afterwards	Prospective evaluation	Risk of death was 56% lower in patients who received EN (HR: 0.44, 95% CI: 0.24, 0.80, *P* = 0.007)
Martin and colleagues [119]	499 ICU patients with an expected ICU stay of at least 48 hours. Introduction of evidence-based recommendations	Cluster-randomized controlled trial	Implementation of evidence-based recommendations led to more days of EN (*P* = 0.042), shorter mean hospital stay (*P* = 0.003) and a trend towards reduced mortality (*P* =0.058). The mean ICU stay did not differ significantly
Doig and colleagues [117]	1,118 patients in the ICU >2 days. Randomization to guideline or control groups. Guideline ICUs used an evidence-based guideline	Cluster-randomized controlled trial	Guideline ICU patients were fed earlier and reached nutritional goals more often compared with control subjects, but did not show significantly different hospital discharge mortality (*P* = 0.75), hospital LOS (*P* = 0.97), or ICU LOS (*P* = 0.42)
Alberda and colleagues [14]	2,772 mechanically ventilated patients. Prescribed and received energy was reported	Observational cohort study	Patients received only 56 to 64% of the nutritional prescription for energy and 50 to 65% for protein. Increased provision of energy and protein appear to be associated with improved clinical outcomes, particularly when BMI <25 or ≥35 kg/m^2^. A 1,000 kcal increase is associated with improved mortality (*P* = 0.014) and more ventilation-free days (*P* = 0.003)
Strack van Schijndel and colleagues [120]	243 sequential mixed medical-surgical patients. Nutrition according to indirect calorimetry and at least 1.2 g protein/kg/day	Prospective observational cohort study	Reaching nutritional goals improves ICU (*P* = 0.027) and 28-day mortality (*P* = 0.005) and hospital survival (*P* = 0.04) in female patients. When only energy goals but not protein goals are met, ICU mortality is not changed. No differences could be observed for male patients
Casaer and colleagues [123]	4,640 ICU patients: 2,312 patients received PN within 48 hours after ICU admission, 2,328 patients received no PN before day 8	Randomized, multicenter trial	Early provision of PN shows a higher complication rate (26.2% vs 22.8% for ICU infections, *P* = 0.008), longer mechanical ventilation time (9.7% longer, *P* = 0.006) and renal replacement therapy (3 days’ longer, *P* = 0.008), and a longer mean hospital duration (6.4% higher likelihood to discharge later, *P* = 0.04), but no significant impact on mortality
Weijs and colleagues [121]	886 mechanically ventilated patients; stratified into three groups: reaching energy and protein target; reaching energy target; and reaching no target	Prospective observational cohort study	Reaching the energy and protein target is associated with a 50% decrease in 28-day mortality. Reaching only the energy target is not associated with an improvement
Arabi and colleagues [124]	240 ICU patients randomly assigned to permissive underfeeding or target feeding	Randomized, controlled trial	Permissive underfeeding may be associated with lower mortality rates. Hospital mortality was lower in the permissive feeding group (30.0% vs 42.5%; relative risk: 0.71; 95% CI: 0.50, 0.99; *P* = 0.04). However, 28-day all-cause mortality was not significantly different between groups (18.3% vs 23.3%; relative risk: 0.79; 95% CI: 0.48, 1.29; *P* = 0.34)
Rice and colleagues [125]	200 mechanically ventilated patients with acute respiratory failure, expected to require mechanical ventilation for at least 72 hours randomized to receive initial trophic (10 ml/hour) or full-energy EN for the initial 6 days	Randomized, open-label study	Mortality to hospital discharge was 22.4% for trophic vs 19.6% for full energy (*P* = 0.62). The trophic group showed a trend for less diarrhea in the first 6 days (19% vs 24% of feeding days; *P* =0.08) and significantly fewer episodes of elevated gastric residual volumes (2% vs 8% of feeding days; *P* <0.001)
Singer and colleagues [126]	130 patients expected to stay in ICU >3 days. Randomization to EN with a target determined by indirect calorimetry (study group) or with 25 kcal/kg/day (control group)	Prospective, randomized, controlled trial	Patients in the study group had a higher mean energy (*P* =0.01) and protein intake (*P* =0.01) than the control group. They also showed a trend towards reduced mortality (32.3% vs 47.7%, *P* =0.058), but the number of infectious complications were higher (37 in the study vs 20 in the control group *P* =0.05)
Allingstrup and colleagues [122]	113 ICU patients. Analyzed according to provided amount of protein and AA	Prospective, observational, cohort study	In the low protein and AA provision group, the Kaplan-Meier survival probability was 49% on day 10, compared with 79% and 88% in the medium and high protein and AA groups on day 10, respectively
Rice and colleagues [127]	1,000 patients with acute lung injury requiring mechanical ventilation. Randomization to trophic or full enteral feeding for the first 6 days	Randomized, open-label, multicenter trial	Initial trophic feeding did not improve 60-day mortality (23.2% vs 22.2%, *P* =0.77) or infectious complications (*P* =0.72, *P* =0.77, and *P* =0.24 for ventilator-associated pneumonia, *Clostridium difficile* colitis and bacteremia, respectively) compared with full enteral feeding
Heidegger and colleagues [128]	ICU patients who had received less than 60% of their energy target from EN, were expected to stay >5 days, and to survive >7 days. Randomization to SPN (*n* =153) or EN (*n* =152). Protein administration was set to 1.2 g/kg ideal bodyweight/day during the study	Randomized controlled trial	Mean energy delivery between days 4 and 8 was higher for the SPN group (103% vs 77% of energy target). Nosocomial infections, between days 9 and 28, were more frequent in the EN group patients (38% vs 27%, *P* =0.0248). Overall nosocomial infections were not different

AA, amino acids; BMI, body mass index; CI, confidence interval; EN, enteral nutrition; FFMc, corrected free fat mass; HR, hazard ratio; LOS, length of stay; N, nitrogen; npo, nil by mouth; PN, parenteral nutrition; SPN, supplementary parenteral nutrition.

Randomized trials focusing only on energy provision have not shown an impact on mortality [123-125,127,129]. Measured energy expenditure may be needed during targeted feeding in the early phase of critical illness to avoid overfeeding, and providing energy at 80 to 90% of energy expenditure may be sufficient [130]. All of the large randomized trials of early PN in critically ill patients have used energy targets based on crude estimations of energy requirements. Hypothetically, a high energy intake may inhibit autophagia, which may be a disadvantage for the critically ill patient, as this enables the accumulation of cellular damage [131]. Whether this inhibition is restricted to patients given PN or whether there is any clinical impact from this observation remains an open question.

No randomized trials to date have adequately studied protein provision in critically ill patients. New studies using whole-body protein kinetics are needed to enable reassessment of the current recommendations [111-113]. Protein intake should be tailored to suit the patient, but presently there is no recognized marker available to guide individualized requirements.

In general, if enteral nutrition is possible, early provision is advantageous to the patient [132] as demonstrated in patients following trauma [133]. In patients with sepsis, the evidence is less robust. However, this evidence assumes enteral nutrition can be delivered to meet both protein and energy goals. Randomized trials have shown that the use of early PN did not improve mortality [123,129]. However, these studies used low protein intake levels compared with current guidelines.

Older studies using the endpoint of N balance or body composition provide less relevant information during the initial phase of critical illness due to the hemodynamic instability of the patient [15,114]. Further research into the relationship between whole-body (and organ) protein turnover and the level of organ failure and energy expenditure is needed to characterize the status of protein metabolism in the early phase of critical illness before any recommendations can be given [31,116,134].

### Route of administration

For healthy individuals, the digestion of food demands energy. However, in terms of N balance there is no difference between enteral and parenteral routes of administration. For the critically ill patient the situation is less clear, as it is difficult to characterize digestion and absorption because patients are rarely in a steady state long enough for N-balance studies to be completed. It should be recognized that AAs provided by PN are free, hydrated molecules, and thus the actual amount of protein substrate provided by parenteral AA mixtures is usually overestimated, the amount provided being approximately 20% less than that provided in food protein [68]. For enteral protein, the level of absorption is a complicating factor because it may be low and/or variable [71].

There is no option to store AAs in the body, so the only way to handle excess protein intake is by AA oxidation. Whether or not the oxidation of AA differs in relation to the route of administration is dependent upon nutritional status, the amount of energy, protein, and AA given, and the level of metabolic stress imposed on the subject. Additional studies are needed in critically ill patients to assess gastrointestinal uptake of AAs and to measure whole-body (and organ) protein turnover and AA oxidation for different levels of intake [31,116,134].

## Conclusions

Nutrition support in the critically ill has to date focused on adequate provision of energy to the patient. Protein and AA provision has been dealt with as a subcomponent of energy supply. However, proteins and AAs are fundamental to recovery and survival, not only to preserve active tissue (protein) mass but also to maintain a variety of other essential functions. The scientific recognition of the importance of protein is growing [68], and although optimal protein dosing studies are not available, expert opinion supports administering in excess of 1.2 g/kg/day [121,122,130]. The use of one fixed protein-to-energy ratio to achieve both energy intake and protein intake targets often results in protein underfeeding or energy overfeeding. A mixed approach with a range of enteral or parenteral formulas may therefore help to balance protein and energy targeted feeding [121,130,135,136]. The identification of a target for protein provision for individual patients is a crucial step in recognizing the key role of protein in nutrition support, especially for obese and older patients (with low muscle mass) who are seen in increasing numbers in the ICU. Further research is urgently needed to assess the specific quantitative and qualitative requirements of these patient subgroups.

## Abbreviations

AA: amino acid
CT: computed tomography
LBM: lean body mass
N: nitrogen
PN: parenteral nutrition
